# Fly Fights with Both Hands

**DOI:** 10.1371/journal.pbio.0020313

**Published:** 2004-09-07

Defending against attack is one of the most important challenges facing any organism. But while sticks and stones may break the bones of a lion, microscopic threats such as bacteria require different weapons. And it's not just we humans who have this problem—insects are prey to bacterial infections too. Their immune systems, however, rely on a far simpler set of defenses than those found in mammals. Exactly how one insect immune system recognizes bacteria, and how it fights off the invader, is the subject of a new study in this issue by Johann Deisenhofer and colleagues.

The fruitfly, Drosophila, has long been known to use a set of molecular sentries called “peptidoglycan recognition proteins,” or PGRPs, that circulate in the fly's bloodstream. When a PGRP recognizes a bacterial invader, it triggers a cascade of events whose ultimate product is a group of antimicrobial compounds that attack and kill the bacteria.

While the family of PGRPs has been extensively studied, exactly how they recognize their target bacteria has been less clear. At the cellular level, recognition requires contact, and the part of the bacterium the PGRP recognizes is, as its name implies, the peptidoglycan. A peptidoglycan is a special sort of molecular polymer found primarily on bacterial cell walls. Peptidoglycan forms when chains of sugar molecules (the glycans) are cross-linked by amino acids (the peptides) to form a meshwork that helps keep the bacterium from bursting under the osmotic strain of its contents.

There are several types of peptidoglycans that differ in their precise sugar and amino acid constituents and in their ability to trigger the Drosophila defensive reaction. Deisenhofer and colleagues set out to determine whether this difference in triggering ability of particular peptidoglycans was linked to differences in the PGRPs that recognize them. To do this, they determined the three-dimensional structure of one PGRP, called PGRP-SA. They worked out not only the overall shape of PGRP-SA, but also which amino acids sat where on the convoluted surface of the protein.

What they found on that surface was an extended groove down one entire side of the protein. To test whether this groove was indeed the recognition site for peptidoglycan, the group introduced a series of mutations to critical amino acids along the groove, testing each new form for its ability to bind peptidoglycan. Indeed, the binding and defense-triggering ability was worse for almost every mutant, demonstrating conclusively that the normal protein uses the groove to bind and recognize peptidoglycan.

Then the team made a surprising discovery. They found that when PGRP-SA comes in contact with bacterial peptidoglycan, it begins to cleave the links between amino acids in the peptide portion of the peptidoglycan. This in itself is not so amazing—animals make plenty of peptide-cleaving proteins. But this protein has a difference, one which makes it unique in the animal kingdom.[Fig pbio-0020313-g001]

**Figure pbio-0020313-g001:**
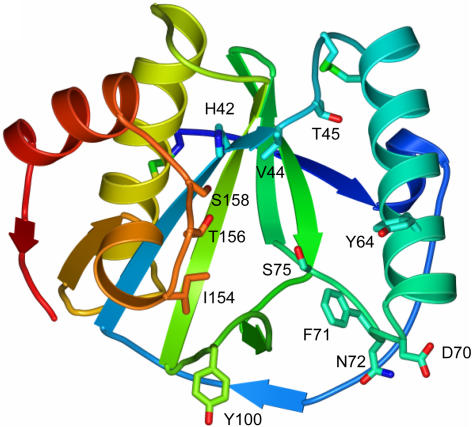
Stick model of the PGRP-SA residues chosen for mutational analysis

To understand this difference, consider your two hands. They are mirror images of each other, alike yet not the same. No amount of twisting and turning will allow you to superimpose one exactly on the other—if you align the fingers, the knuckles will point in opposite directions, and if the knuckles point the same way, the fingers are all mismatched. This type of relationship between mirror images, called chirality (from the Greek for “hand”), is found in amino acids as well, a result of the three-dimensional geometry that radiates from their central atom.

All the amino acids used by all known animal species are exclusively of the “left-handed” form, and the protein-digesting enzymes we make are designed specifically for these L-amino acids. Bacteria, however, link left-handed and right-handed amino acids together to form peptidoglycan. What Deisenhofer's team discovered was that unlike any other known animal enzyme, the Drosophila PGRP-SA was able to break apart this “L,D” (levo-dextro) linkage, making it, in their words, “the first eukaryotic protein exhibiting such an activity specific for peptide bonds existing only in prokaryotes.”

What does it all mean? Deisenhofer and colleages' results are yet another demonstration that at the molecular level, understanding structure is the key to understanding function. They also show that when it comes to defense, it helps to be able to fight with both hands.

